# Description of the Microsporidian Parasite, *Heterosporis sutherlandae* n. sp., Infecting Fish in the Great Lakes Region, USA

**DOI:** 10.1371/journal.pone.0132027

**Published:** 2015-08-05

**Authors:** Nicholas B. D. Phelps, Sunil K. Mor, Aníbal G. Armién, Katharine M. Pelican, Sagar M. Goyal

**Affiliations:** 1 Veterinary Diagnostic Laboratory, College of Veterinary Medicine, University of Minnesota, 1333 Gortner Avenue, Saint Paul, Minnesota, 55108, United States of America; 2 Veterinary Population Medicine Department, College of Veterinary Medicine, University of Minnesota, 1365 Gortner Avenue, Saint Paul, Minnesota, 55108, United States of America; Illinois Institute of Technology, UNITED STATES

## Abstract

Heterosporosis is an increasingly important microsporidian disease worldwide, impacting wild and farmed raised fishes in both marine and freshwater environments. A previously undescribed species (*Heterosporis* sp.), with widespread distribution in the Great Lakes region, was the subject of this study. Three angler-caught fish were submitted to the Minnesota Veterinary Diagnostic Laboratory from 2009–2010 with lesions caused by intracellular proliferation of parasitic spores, resulting in destruction and eventual widespread necrosis of the host skeletal muscles. Mature ovoid (5.8 x 3.5μm) spores of a microsporidian parasite, consistent with the genus *Heterosporis*, were observed by light and electron microscopy. Molecular identification was performed using primer walking to obtain a near-complete rRNA gene sequence (~3,600 bp). A unique species of *Heterosporis* was identified, demonstrating less than 96% sequence identity to other published *Heterosporis* sp. on the basis of partial rRNA gene sequence analysis. *Heterosporis sutherlandae* n. sp. (formerly *Heterosporis* sp.) was identified in yellow perch (*Perca flavescens*), northern pike (*Esox lucius*) and walleye (*Sander vitreus*) from inland lakes in Minnesota and Wisconsin. Previous research suggests this species may be even more widespread in the Great Lakes region and should be reexamined using molecular techniques to better understand the distribution of this novel species.

## Introduction

Microsporidian parasites infect a wide range of hosts, from single-celled protozoa to higher vertebrates, including humans. The first microsporidium recorded in vertebrates was *Glugea anomala*, producing subcutaneous cysts in the three-spined stickleback (*Gasterosteus aculeatus*) [1]. Now, more than 100 species of microsporidia in 15 genera have been identified in fishes [1–3]. Many of the microsporidia that infect fish are important parasites and in chronic infections, they may cause reduced growth, anorexia, impaired swimming ability, reproductive defects and liquefaction of muscle tissues [4–11].

Members of the genera *Heterosporis*, *Dasyatispora*, *Pleistophora*, *Kabatana*, and the species in the undescribed group microsporidium are known to infect the skeletal muscles of susceptible fish hosts [3, 12–14]. Fish are exposed to the parasitic spores by consumption of an infected host or spores in the water column. As the disease progresses, muscle cells are destroyed and replaced by connective tissue, resulting in an emaciated or concave appearance [14, 15]. The damage caused by the parasite gives filleted meat a freezer-burn appearance and renders the fish unfit for human consumption [15, 16]. Although direct mortality of the host has been suggested in extreme cases [14], indirect parasite-induced mortality due to failure to capture prey or escape predation is likely.

The genus *Heterosporis* includes seven recognized species infecting fresh and saltwater fish of Africa, Europe (*H*. *finki*, *H*. *cichlidarum*, *H*. *schuberti* [17, 18]), Japan, Taiwan (*H*. *anguillarum* [15, 19]), USA (*Heterosporis* sp. [20]), and the Arabian Gulf (*H*. *saurida* [14]). The molecular phylogeny of the rRNA gene divided fish-infecting microsporidia into five groups (group 1–5) with *Heterosporis* falling into group three [3]. The characteristic feature of the genus *Heterosporis* is the presence of a dense solid wall sporophorocyst, which encloses all developmental stages (meronts, sporonts, sporophorous vesicles with sporoblasts, and spores) of the parasite as observed under an electron microscope [3].

An undescribed species, *Heterosporis* sp., from the USA was the subject of this investigation. This parasite was first detected in 2000 by Sutherland et al. [20] and D. Cloutman (personal communication) in skeletal muscles of yellow perch (*Perca flavescens)* in Wisconsin and Minnesota, respectively. This parasite has been reported from 26 bodies of water in Minnesota, 16 in Wisconsin, 2 in Michigan, and 1 in Ontario (personal communication with respective state agencies). Susceptible fish species, on the basis of natural infections or laboratory trials, include a number of economically and ecologically important fish such as yellow perch, walleye (*Sander vitreus*), northern pike (*Esox Lucius*), rainbow trout (*Oncorhynchus mykiss*), channel catfish (*Ictalurus punctatus*), largemouth bass (*Micropterus salmoides*), fathead minnow (*Pimephales promelas*), and koi (*Cyprinus carpio koi*) [16]. *Heterosporis* sp. has been listed as a reportable pathogen or a disease of concern in many states including Illinois, Maine, Michigan, Minnesota, Utah, and Wisconsin (personal communication with respective state agencies) and has been identified as a disease of concern by the Great Lakes Fisheries Commission. The following description of the previously undescribed species of *Heterosporis* is based on morphologic characteristics and phylogenetic analysis.

## Materials and Methods

### Ethics statement

The samples used in this study were submitted to the Minnesota Veterinary Diagnostic Laboratory for disease diagnosis and therefore no IACUC approval was needed. Archived samples were obtained from P.E. Miller [16], who conducted all procedures under approval from UW-La Crosse Institutional Animal Care and Use Committee (IACUC) [16].

### Sample source

Three fish submitted from 2009–2010 to the Minnesota Veterinary Diagnostic Laboratory (MVDL; St. Paul, Minnesota) and suspected of being infected with *Heterosporis* sp. were examined in this study (Table 1). The fish were angler-caught by hook and line and sent to the Minnesota Department of Natural Resources (MDNR; St. Paul, Minnesota) or directly to the MVDL. Whole fish were transported overnight on ice packs to the laboratory. At the laboratory, fish were immediately examined or held at 4°C for no more than 24 h. All samples were examined by standard diagnostic tests consisting of visual inspection of muscle tissue and wet mount by light microscopy. In addition, three archived *Heterosporis* sp.-positive muscle samples were submitted to the MVDL from the US Fish and Wildlife–La Crosse Fish Health Center (La Crosse, Wisconsin). These samples came from experimentally infected fathead minnows that were fed infected muscle tissue of yellow perch from Catfish Lake, Villas County, Wisconsin [16].

**Table 1:** *Heterosporis*-positive fish submitted to the Minnesota Veterinary Diagnostic Laboratory from 2009–2010. All samples were confirmed by gross lesions, light microscopy, and sequence analysis. Fish were collected by hook and line recreational fishing.

Host Species	Body of water	Location	Comments
Walleye	Lake Wabana	Itasca County, MN	First report in this body of water
Yellow perch	Leech Lake	Cass County, MN	Angler reported 30% of fish were suspect positive
Northern pike	Unknown	MN	Unknown source
Yellow perch	Catfish Lake	Villas County, WI	Archived sample from experimental infection

### Light microscopy

Infected and uninfected samples of skeletal muscle and major organs (kidney, liver, spleen, brain, intestine, and heart) were fixed in 10% neutral buffered formalin and processed for paraffin embedding. Four-micron thick sections were stained with hematoxylin and eosin (H&E). In addition, selected sections were stained with Giemsa, Grocot Metanamine Silver (GMS), Periodic Acid-Shiff (PAS), Gram stain, and acid fast. Some sections were left unstained. All sections were examined under a light microscope to determine susceptibility to the various stains.

### Electron microscopy

Formalin fixed muscle tissue from the infected yellow perch originating from Leech Lake (Cass County, MN) was post-fixed in 0.166 M cacodylate-buffered, 3% glutaraldehyde with 1% tannic acid solution (Electron Microscopy Sciences, Hatfield, Pennsylvania), followed by a second post-fixation treatment in 1% osmium tetroxide (Electron Microscopy Sciences). Using a graded series of ethyl alcohol, 1.0-mm^3^ tissue blocks were dehydrated and embedded in Embed (Electron Microscopy Sciences). Embedded samples were trimmed and sectioned on a Leica UC6 Ultramicrotome (Leica Microsystems, Vienna, Austria). Thin sections (60–90 nm) were collected on 100-mesh copper grids (Electron Microscopy Sciences) and grids were stained with 5% uranyl acetate for 20 minutes and Satos’ lead citrate for 6 min. The sections were examined under a JEOL transmission electron microscope (JEOL Ltd., Tokyo, Japan). Dimension of microorganism structures were taken and analyzed using iTEM software (Olympus SIS, Munster, Germany).

For scanning electron microscopy, purified microsporidian spores from yellow perch were post-fixed in 2.5% glutaraldehyde in 0.1M sodium cacodylate buffer overnight. The sample was washed three times in 0.1M sodium cacodylate buffer and post fixed in 1% osmium tetroxide in 0.1M sodium cacodylate buffer. The sample was rinsed three times in deionized water and passed through a series of ethanol dehydration steps in the following order 25%, 50%, 70%, 95%, and 100%. Following dehydration, samples were placed in a critical point dryer and dehydrated further followed by coating in the sputter coater. Microorganisms were examined on a Hitachi S3500N scanning electron microscope (Tokyo, Japan).

### DNA extraction and PCR amplification

*Heterosporis* DNA was amplified from infected tissues by end-point PCR. Briefly, total DNA was extracted using Qiagen DNeasy Blood and Tissue extraction kit in a final elution volume of 100μl (Qiagen, Valencia, California). Sets of six overlapping primer pairs were used to amplify the entire sequence of the rRNA gene (S1 Table). A 50μl reaction mix was prepared for PCR using 1.5μl of each primer (10pmol/μl), 25μl of HotStar *Taq* master mix (Qiagen), 18μl nuclease-free water, and 4μl of template DNA. The PCR thermal cycling protocol consisted of an initial denaturation at 95°C for 15 min followed by 35 cycles of 1 min at 94°C, 1 min at respective annealing temperatures (S1 Table), 1 min at 72°C and a final elongation for 10 min at 72°C. The PCR product was visualized after 1% agarose gel electrophoresis.

### Sequencing and phylogenetic analysis

The PCR amplicons were purified using a QIAquick PCR purification kit (Qiagen). Each DNA fragment was sequenced twice in both directions using the same forward and reverse primers used in the initial PCR. Sequencing was performed at the University of Minnesota Genomic Center (UMGC; St. Paul, Minnesota). The sequences were assembled using Sequencher 5.1 software (http://genecodes.com) and contiguous sequences were used in subsequent BLASTn searches of the National Center of Biotechnology Information non-redundant nucleotide (nr/nt) database. Comparable sequences identified in GenBank were aligned using the ClustalW utility in MEGA 6.05 [21]. *Loma embiotocia* (AF320310) was chosen as the outgroup. The best substitution model for analysis of DNA sequences was selected on the basis of the lowest BIC score (Bayesian Information Criterion) in MEGA 6.05. The nucleotide substitution model Kimura 2 parameter +G (Gamma distribution with 4 rate categories) was used to generate phylogenetic trees based on the lowest BIC score. In addition, phylogenetic trees were constructed with the nucleotide substitution model GTR (General Time Reversible) +G (Gamma distribution with 4 rate categories) to verify tree topology. The Maximum Likelihood phylogenetic trees were statistically validated using 1,000 bootstrap replicates [22]. The nucleotide percent identities and histogram were constructed using Geneious Pro [23].

## Results

### Gross and light microscopy

**Fig 1:**
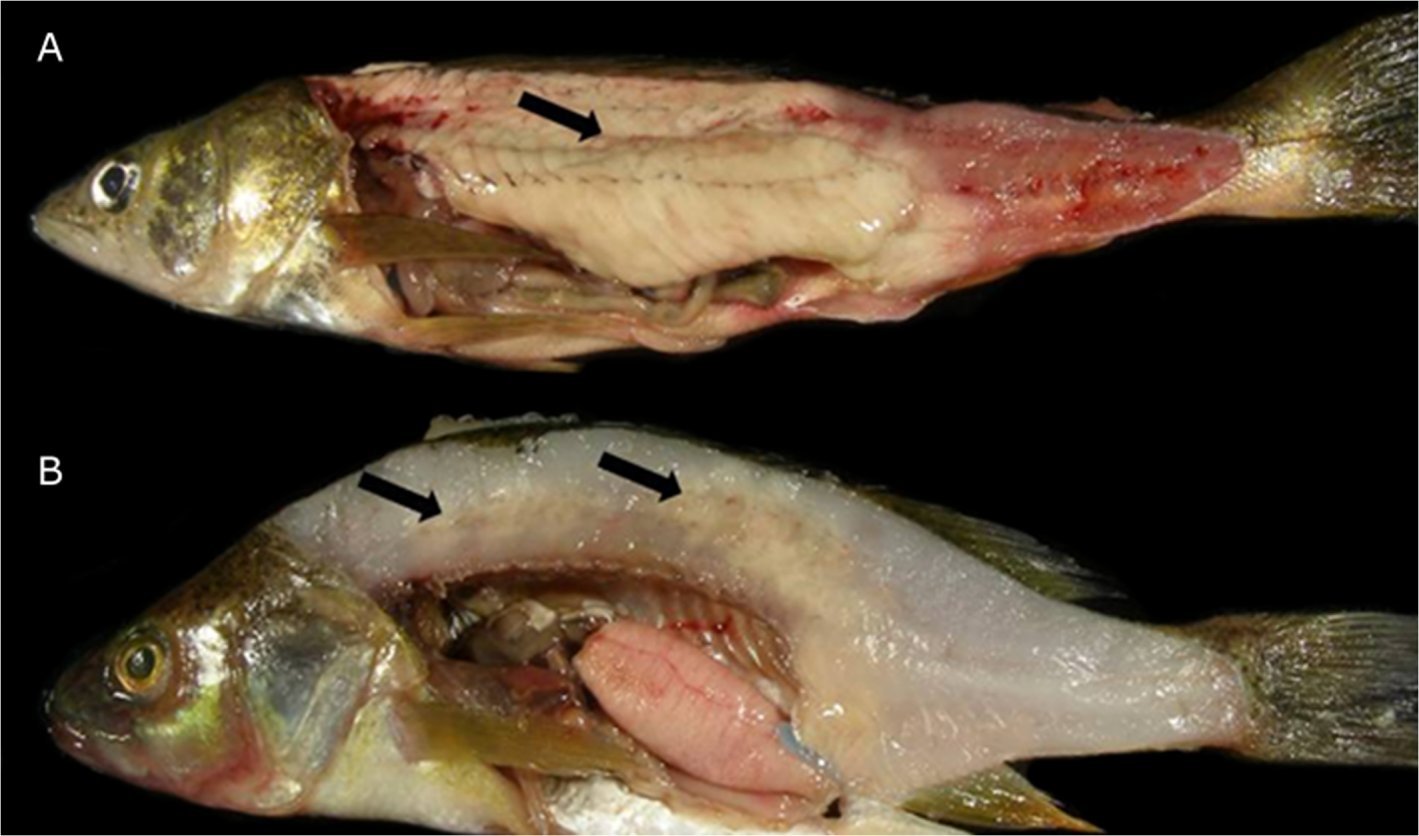
*Heterosporis*-positive fish submitted to the Minnesota Veterinary Diagnostic Laboratory from 2009–2010. **A)** Walleye (*Sander vitreus*) 29.2 cm long. **B)** Yellow perch (*Perca flavescens*) 16.3 cm long. Arrows indicate sites of *Heterosporis*-induced lesions, with multifocal to coalescing necrosis of muscle tissue.

**Fig 2:**
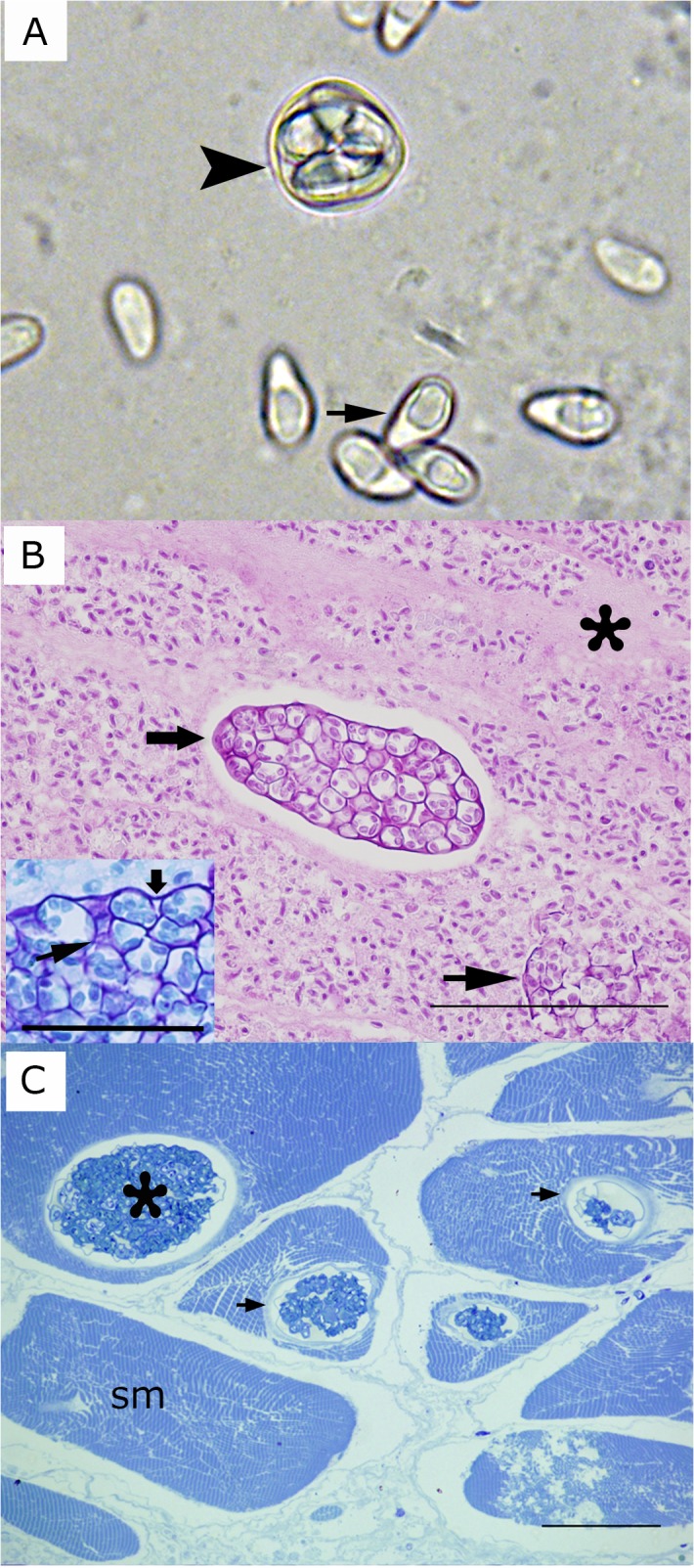
**A)** Fresh preparation of microsporidia-infected fish. Arrow indicates a spore of *Heterosporis sutherlandae* n. sp. Arrowhead shows a sporophorous vesicle with several spores. **B)** Widespread muscle destruction due to *H*. *sutherlandae*. Mature spores have replaced muscle cells and are surrounded by loose fibrous tissue (asterisk). The wide arrow indicates a large sporophorocyst containing sporoblast and spores. The narrow arrow shows ruptured sporophorocyst vesicles. Tissue embedded in paraffin and stained with PAS. Scale bar is 100 μm. Inset: Giemsa stained preparation showing a detail of the wall of a sporophorocyst (wide arrow) and the wall of a sporophororous vesicle (arrowhead). Scale bar is 25 μm. **C)** Sporophorocysts (asterisk) within skeletal muscle cells (sm). Arrows indicate the sporophorocystic wall. Tissue embedded in resin and stained with Toluidine blue. Scale bar is 12 μm.

Gross lesions were characterized as multifocal to coalescing to locally extensive necrosis of skeletal muscle tissue (Fig 1A and 1B). The distribution and severity of *Heterosporis*-induced lesions varied among samples. In fresh preparations, spores were oval to pyriform with an eccentrically located posterior vacuole (Fig 2A) and measured 5.8 ± 0.5 μm (4.8–6.3 μm) × 3.45 ± 0.15 (3.2–3.6 μm). Occasionally, spores were inside sporophorous vesicles, typically containing 4–8 spores (Fig 2A). The muscle fibers were distended and sarcoplasm and myofibrils were replaced with large numbers of *Heterosporis* spores at different stages of development. In areas of high spore concentration, a majority of the skeletal muscle cells were replaced by the parasites (Fig 2B). In less affected areas of the skeletal muscle, sporophorocysts containing developmental stages were found (Fig 2B). There was a multifocal granulomatous inflammatory infiltration expanding the interstitium of the skeletal musculature. Macrophages were distended and contained numerous developing spores within phagocytic vacuoles. A mild amount of fibrous tissue replaced necrotic skeletal muscles fibers. Despite the advanced infection of skeletal muscle tissue, no spores were observed in histological sections of kidney, liver, spleen, brain, intestine, and heart. In addition to H&E, Giemsa and PAS were also demonstrated to have diagnostic value since parasites and cyst membranes were clearly identified when stained (Fig 2B and inset).

### Electron microscopy

**Fig 3:**
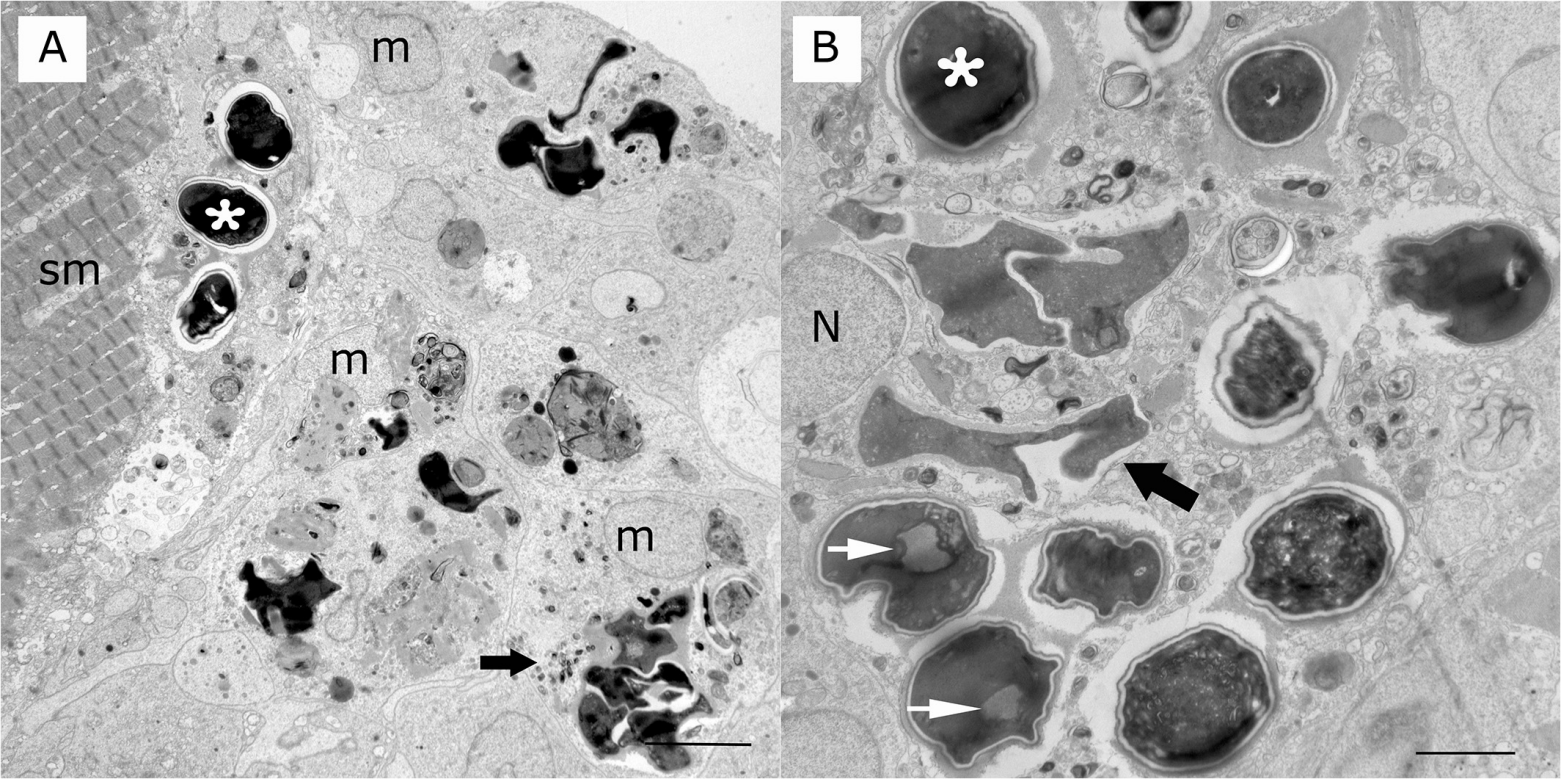
Transmission electron microscopy of microsporidian granulomatous myocytis in a yellow perch. **A)** Multiple mature spores (asterisk) at various stages of digestion (wide arrow) and cell and parasitic debris inside macrophages (m) and a muscle cell (sm). Scale bar is 5 μm. **B)** Multiple mature spores (asterisk) at various stages of digestion are inside parasitophorous vacuoles and phagolysosomes of macrophages (wide arrow). Spores display a spore wall and posterior vacuoles (white arrow). Nucleus (n) of macrophage. Scale bar is 5 μm.

**Fig 4:**
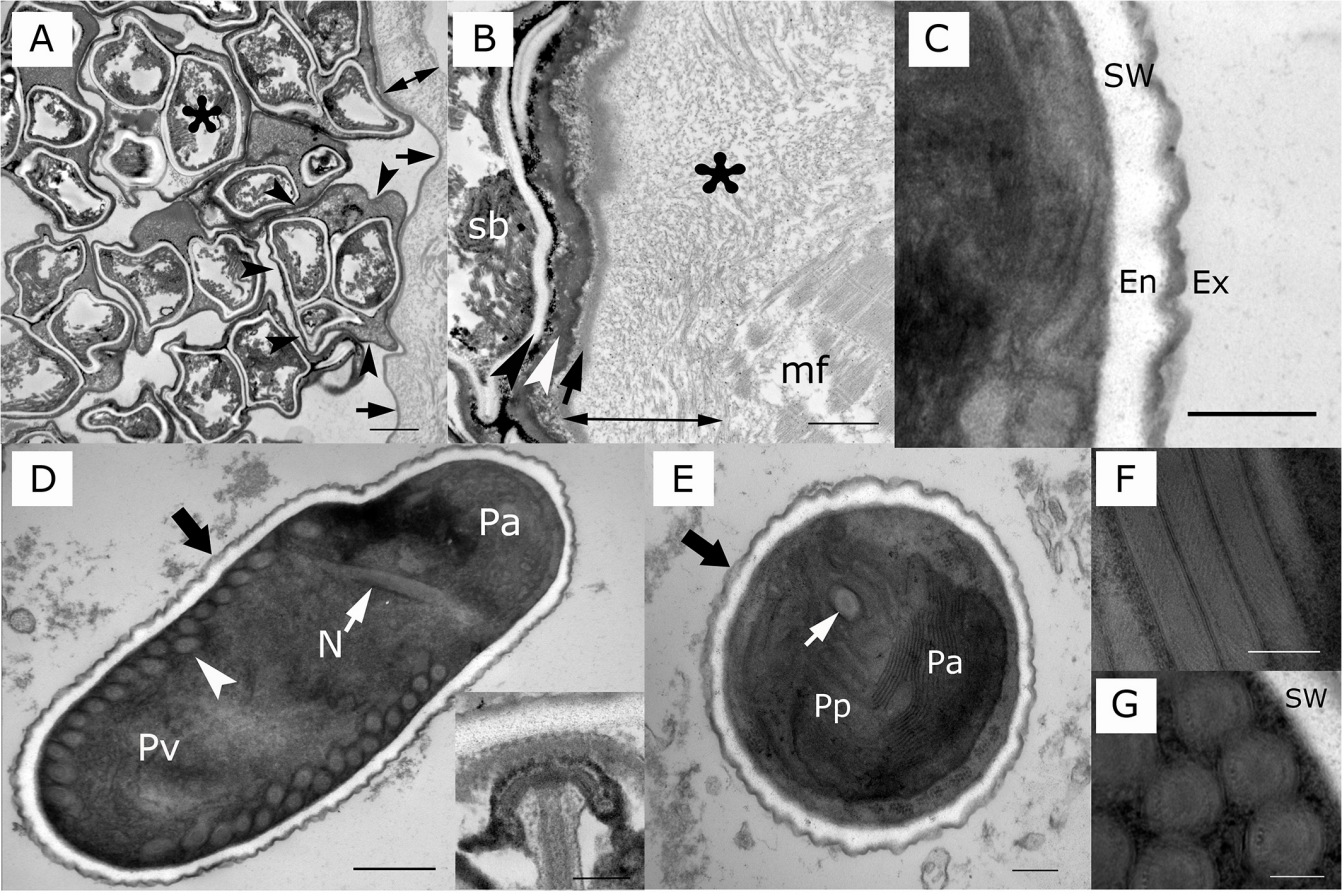
*Heterosporis sutherlandae* n. sp. from yellow perch and walleye. **A)** Sporophorocyst containing several sporophorous vesicles. The sporophorocyst is lined by a thick wall (double headed arrow). The inner surface of the sporophorocyst is indicated by arrows. The edge of the wall of one sporophorous vesicle is highlighted by arrowhead. Spore is marked by an asterisk. Scale bar is 2 μm. **B)** Magnification of the sporophorocyst wall (double headed arrow) in a muscle fiber (mf); note the thick external layer, which is formed of microtubules and filaments (tf) and an inner electron dense membrane (arrow). The most internal smooth electron dense layer is a sporophorous vesicle, which is formed of an electron dense amorphous material (white arrowhead). A sporoblast (sb) is near the wall of the sporophorous vesicle. Scale bar is 1 μm. **C)** Spore wall (sw) of 122–137 nm comprised of an electron dense exospore (ex) measuring 20.78–33.98 nm and an electron lucent endospore (en) measuring 101.76–103.36 nm. Scale bar is 200 nm. **D)** Longitudinal section of *H*. *sutherlandae* displaying a spore wall (wide arrow), posterior vacuole (Pv), coiled filament (white arrowhead), polar filament (white arrow), nucleus (N), and anterior polaroplast. Scale bar is 0.5 μm. Inset: Anchoring disk of a spore. Scale bar is 200 nm. **E)** Transverse section of the anterior pole of *H*. *sutherlandae* displaying a spore wall (wide arrow), polar filament (white arrow), and anterior (Pa) and posterior polaroplast (Pp). Scale bar is 200 nm. **F)** Longitudinal section of coiled filament of *H*. *sutherlandae*. Scale bar is 200 nm. **G)** Transverse section of polar filaments of *H*. *sutherlandae* showing a concentrically multilayer structure. Spore wall (sw). Scale bar is 200 nm.

On toluidine blue stained plastic embedded sections, *Heterosporis* sp. was observed in the muscle tissue of the walleye and yellow perch. Spores were found free in the interstitium admixed in cell debris, within muscle fibers inside of sporophorocysts (Fig 2C) and within phagocytic vacuoles of macrophages (Fig 3A and 3B). Large numbers of spores within macrophages were in different stages of degradation (Fig 3A and 3B). A few spores were present in the subsarcolema (Fig 3A). Sporophorocysts containing all developmental stages within sporophorous vesicles were observed in both walleye and yellow perch tissues (Fig 4A). However, very few sporophorous vesicles containing meronts stage were observed. The sporophorocystic wall derived presumably from the host cell was compounded with one thick layer of different electron densities. The outermost interface was composed of packed intermediary filaments, microtubules and cisternae from the endoplasmic reticulum (Fig 4A and 4B). Lining the innermost surface of the sporophorocystic wall was an electron dense smooth layer (Fig 4B). A sporophorocyst contained a few to large numbers of sporophorous vesicles, which had an average of 4–8 maturating spores with a maximum of 13 (Table 2). These were embedded in an electron opaque matrix.

**Table 2:** Comparison of *Heterosporis* species infecting fish.

Species	Host	Site of infection	Spore shape	Spore size in μm (Polar tube coils)		Number of spores per sporophorous vesicle		GenBank accession	rDNA sequence in GenBank (bp)	Reference
				Mi*	Ma*	Mi	Ma			
*Heterosporis finki*	*Pterophyllum scalare*	Connective tissues, cells, myocytes	Ovoid elongated posterior flat	3.0 x 1.5 (8)	8.0 x 2.5 (30–36)	16+	8	-	-	[17]
*Heterosporis cichlidarum*	*Hemichromis bimaculatus*	Gill filaments	Ovoid, slightly pyriform	-	7.0–8.0 x 4.0–4.5 (30–39)	-	10–12	-	-	[18]
*Heterosporis schuberti*	*Ancistrus cirrhosis*, *Pseudocrenilabrus multicolor*	Skeletal tissue	Ovoid	3.4–4.9 x 2.4–3.4 (-)	5.4–8.8 x 2.9–4.9 (40–42)	26	4–16	-	-	[15]
*Heterosporis anguillarum*	*Anguilla japonica*	Skeletal tissues	Elongated ovoid	3.5 x 2.4 (33–36)	7.8 x 4.5 (33–46)	-	-	AF402839	4,060	[15, 19]
*Heterosporis saurida*	*Saurida undosquamis*	Skeletal muscles, mesenteric tissues	Ovoid to pyriform	3.3 x 2.0 (5–6)	5.6 x 3.3 (20–21)	-	-	JF745533	~1,100	[14]
*Heterosporis* sp.	*Perca flavescens*, *Esox lucius*, *Sander vitreus*	Skeletal muscles, mesenteric tissues	Ovoid to pyriform	-	5.8 x 3.5 (18–21)	-	4–8	KC137548 to KC137550	3,646	This study

*Mi = microspore; Ma = macrospore

The wall of the sporophorous vesicles was composed of an electron dense and smooth membrane derived presumably from the parasite (Fig 4B). The spore wall consisted of an outer layer (the exospore), which is thinner, electron dense and has a smooth surface. The endospore, on the other hand, is much ticker and electron lucent (Fig 4C). The total thickness of the spore wall was 122.54 to 137.34 nm (Fig 4C). The spore contents consisted of extrusion apparatus, a sporoplasm with a single nucleus, and a vacuole located at the posterior end (Fig 4D). The content of the posterior vacuole varied; in some spores, it contained variably dense amorphous materials but in others, it was empty (Fig 4B and 4D). The polaroplast consisted of a region of closely and loosely packed series of membranes (Fig 4D and 4E). The anchoring disk was located at the spore apex (Fig 4D inset). The number of coils of the polar tube were 18 to 21 arranged in a single row along the inside periphery of the spore (Fig 4D, Table 2). In cross sections, the coiled polar filament measured 133.58 to 163.95 nm in diameter and appeared to be divided into three different concentric layers of variable electron densities (Fig 4F and 4G). On scanning electron microscopy, the spores were round to oval (picture non-shown).

### Genetic analysis

**Fig 5:**
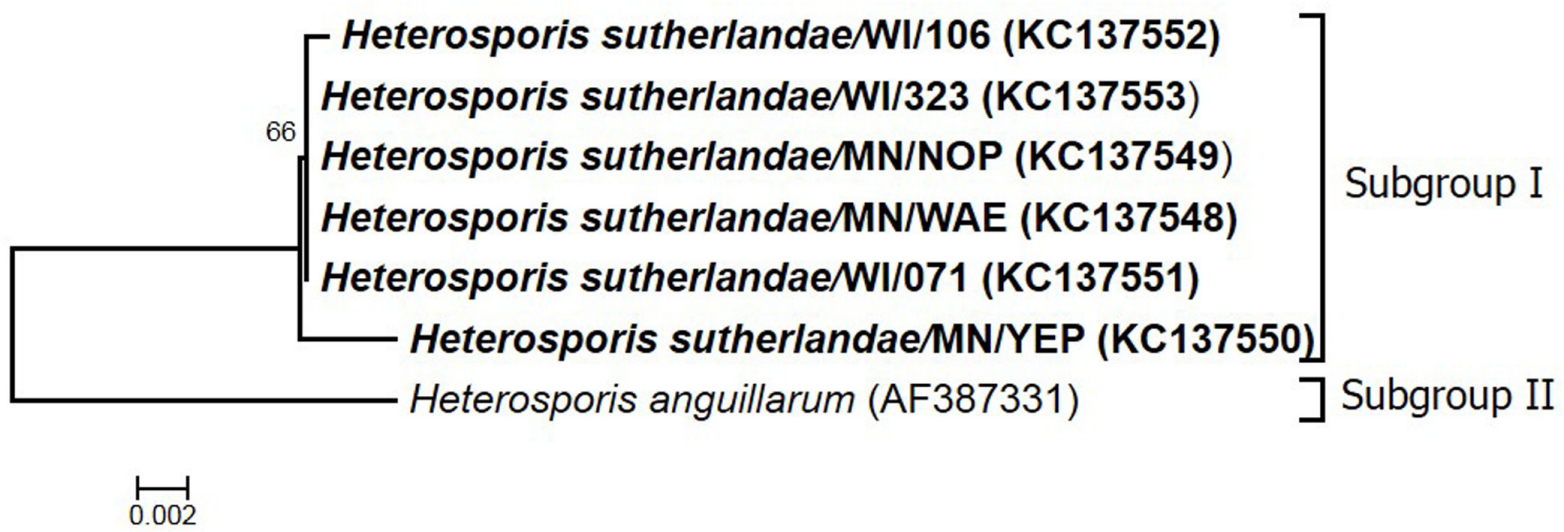
Phylogenetic analysis based on the 3,646bp nearly complete ribosomal RNA gene of *Heterosporis* sp., corresponding to nucleotide positions 170 to 3,815 of reference sequence AF387331. The Maximum Likelihood phylogenetic tree was constructed using the General Time Reversible (GTR) model of nucleotide substitution (4 gamma categories) with 1,000 bootstrap replicates.

The tree topology was the same with both nucleotide substitution models; here we describe phylogenetic trees constructed by the GTR+G model. Phylogenetic analysis was based on a 3,646bp segment of the rRNA gene, corresponding to nucleotide positions 170 to 3,815 of the related species, *H*. *anguillarum* (AF387331). Based on the phylogenetic analysis, *Heterosporis* sp. sequences grouped together in one clade with 99.6%–100% sequence identity with each other, and 97.0%-97.5% sequence identity with *H*. *anguillarum* forming a separate clade (Fig 5). The insertion of nucleotides in each segment of the rRNA gene (SSU, ITS and LSU), along with nucleotide changes, accounted for the variation between *Heterosporis* sp. and other *Heterosporis* species (S1 Fig).

**Fig 6:**
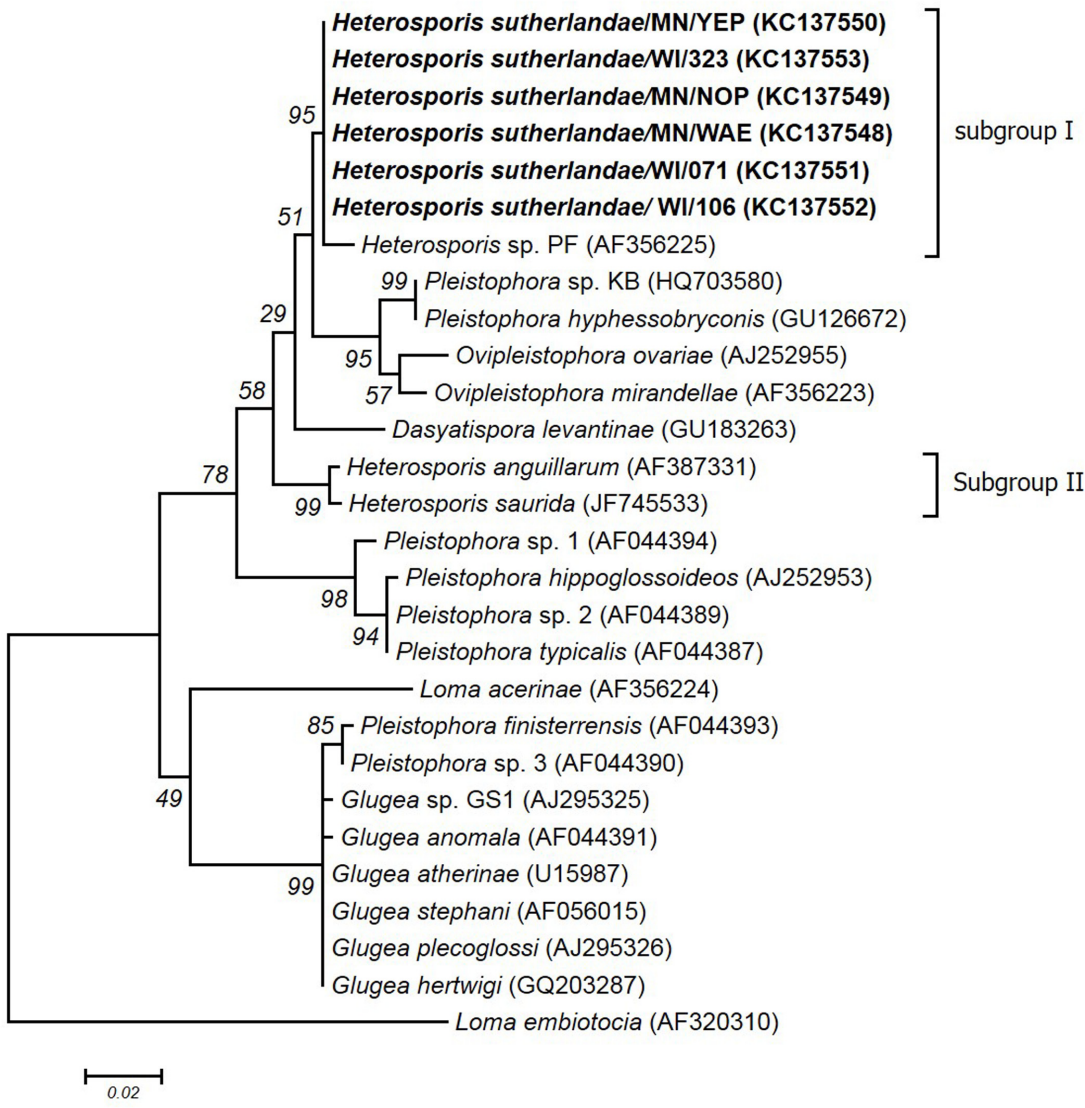
Phylogenetic analysis based on a 1,200 bp nucleotide sequence, corresponding to nucleotide positions 600 to 1,800 of reference sequence AF387331. The Maximum Likelihood phylogenetic tree was constructed using the General Time Reversible (GTR) model of nucleotide substitution (4 gamma categories) model with 1,000 bootstrap replicates.

Most of the fish-infecting microsporidian sequences in GenBank are partial sequences of the SSU, complete ITS, and partial LSU of the rRNA gene. Therefore, additional phylogenetic analysis was performed on a 1,200 nucleotide segment (nucleotide position 600 to 1,800 of *H*. *anguillarum*). Based on this phylogenetic analysis, *Heterosporis* species were divided into two subgroups (I and II) within group 3 of the fish-infecting microsporidia (Fig 6). Subgroup I included six sequences from this study (*Heterosporis* sp.), as well as the previously unclassified *Heterosporis* sp. (AF356225) first reported in Wisconsin [20]. Subgroup II included *H*. *saurida* (JF745533) and *H*. *anguillarum* (AF387331). Subgroup I sequences have 98.5%–100% sequence identity within the subgroup and 92.4%–96.0% sequence identity with subgroup II (S2 Table). Subgroup I was also closely related to *Ovipleistophora mirandellae* (AF356223), *Ovipleistophora ovariae* (AJ252955), and *Pleistophora hyphessobryconis* (GU12667) having 93.8–94.1%, 92.1–94.1%, and 94.5%-94.8% sequence identity, respectively (S2 Table).

### Description of *Heterosporis sutherlandae* n. sp.

#### Systematic position.

Family: *Glugeidae* (Thelohan, 1892)
Genus: *Heterosporis* (Schubert, 1969)
Species: *Heterosporis sutherlandae*

#### Description of species.

Type host: Yellow perch (*Perca flavescens*)
Type locality: Leech Lake, Cass County, Minnesota
Site of infection: Skeletal muscle

Etymology: Species name represents Dr. Daniel Sutherland, who did much of the early work on *Heterosporis* sp.

Genetic sequence: All sequences generated in this study were submitted to GenBank with accession numbers KC137548 to KC137550 for *H*. *sutherlandae/*MN/WAE, *H*. *sutherlandae*/MN/NOP, and *H*. *sutherlandae*/MN/YEP; and KC137551 to KC137553 for *H*. *sutherlandae*/WI/071, H. *sutherlandae*/WI/106, and H. *sutherlandae*/WI/323, respectively.

## Discussion

This study of a previously unclassified microsporidian parasite identified a novel species of *Heterosporis*, *H*. *sutherlandae*, infecting fish in the Great Lakes region. The clinical lesions, morphology and phylogenetic analyses were consistent with other species of this genus. *Heterosporis sutherlandae* was characterized based on the nearly complete rRNA sequence and was indistinguishable from *Heterosporis* sp. reported by Sutherland et al. [20] and Miller [16]. *Heterosporis sutherlandae* formed a separate grouping from two previously known *Heterosporis* sequences: *H*. *anguillarum* reported from Japanese eel *Anguilla japonica* in Japan and Korea and *H*. *saurida* from lizardfish *Saurida undosquamis* in Arabian Gulf region in Saudi Arabia [14, 24, 25] indicating geographical variations of *Heterosporis* sequences. Only a limited number of sequences are currently available in GenBank for comparison and hence there is a need for further molecular epidemiology studies to understand the prevalence and circulation of different species of *Heterosporis*. The insertions, deletions, and substitutions of nucleotides in the SSU, ITS and LSU regions of the complete rRNA may serve as useful markers to differentiate various *Heterosporis* species.

Similar to microsporidia species in *Dasyatispora*, *Pleistophora*, *Kabatana*, the undescribed group microsporidium, and other species in *Heterosporis*, *H*. *sutherlandae* is a microsporidium that causes severe infection of the skeletal muscle with degradation and lysis of the sarcoplasm by mature spores, which places them within the group of non xemoma-forming microsporidia of fish [3, 12–14]. All stages of the life cycle were present in sporophorocysts. A few meronts were found in extensive samples examined and their description remains a subject for future experimental infection or *in vitro* investigation.

In general, the spore structure does not differ among microsporidia genera [3]. The major differences between *H*. *sutherlandae* and other members of the genus *Heterosporis* that infect fish are a variation of the number of coils in the polar tube of mature spores, the formation of macro- and microspores, and the number of mature spores in sporophorous vesicles. *Heterosporis sutherlandae* has 18–21 turns in the polar tube, which is most similar to the macrospores of *H*. *saurida*, which has 20–21 turns [14]. Macro- and microspores of *H*. *sutherlandae* were not observed in fresh preparations or embedded infected tissues. Except for *H*. *cichlidarum*, other species of the genus *Heterosporis* do produce macro- and microspores, which differ in size and number of polar filament coils [3, 18]. Despite the similarity of mature spores, those of *H*. *cichlidarum* are larger, contain more polar tube coils, and are more numerous inside sporophorous vesicles than *H*. *sutherlandae*.

The disease reported here, and those previously reported in the Great Lakes region (Michigan, Minnesota, Ontario, and Wisconsin) as *Heterosporis* sp., was due to intracellular proliferation of spores, resulting in destruction and eventual widespread necrosis of the host skeletal muscle tissue. These reports are likely due to the same or closely related species of *Heterosporis*. As part of this study, *H*. *sutherlandae* was confirmed in three Minnesota lakes (Leech Lake, Lake Wabana, and an unknown lake) and a Wisconsin lake (Catfish Lake). This is a first report of heterosporosis in Lake Wabana. The origin of *H*. *sutherlandae* in the Great Lakes region is currently unknown; however, the characterization of additional samples and related species will help to better understand the phylogeography and evolution of this important parasite. Given the close proximity of water bodies and many potential routes of transmission (i.e. boat traffic, baitfish, etc) the disease could spread to naïve populations. To date, all *Heterosporis*-positive locations have been based on the observation of clinical muscle lesions. No reports of screening of apparently healthy and potentially subclinical fish for the presence of *Heterosporis* are available. Surveillance by sensitive molecular methods is warranted to assess the distribution, risk of spread, and evolution of this important parasite.

*Heterosporis sutherlandae* poses a significant threat to farmed fish with the loss of marketable product due to the destruction of muscle tissue in infected fish. Once established in a farm, the lack of therapeutics and resistant nature of the spores would make eradication and management of the disease difficult. However, the effects of *H*. *sutherlandae* on the population dynamics and catchable biomass of wild fishes are not well understood. To date, no population level affects have been conclusively attributed to heterosporosis in the Great Lakes region. However, the potential indirect parasite-induced mortality, added stress in an increasingly compromised ecosystem, and loss of filet quality for harvest is of concern. Further research into the effects of this parasite on the host and population levels is needed to inform evidence-based management decisions.

## Supporting Information

S1 Fig Comparison of nucleotide sequences of partial SSU, complete ITS and partial LSU regions (600 to 1,800 nucleotides of reference sequence AF387331) of nine *Heterosporis*. First position in image represents 600^th^ nucleotide position of the reference sequence.
(PDF)

S1 Table Primers sets and corresponding annealing temperatures used for DNA amplification and sequencing. (DOCX)

S2 Table Percent identities of *Heterosporis sutherlandae* isolates with previously reported fish-infecting microsporidia. (DOCX)
