# Supplementary material for: Description of the Microsporidian Parasite, Heterosporis sutherlandae n. sp., Infecting Fish in the Great Lakes Region, USA
Source: PLoS One. 2015 Aug 5;10(8):e0132027. doi: 10.1371/journal.pone.0132027 (PMC4526549; doi:10.1371/journal.pone.0132027)
Supplement: S1 Table — (DOCX) [file pone.0132027.s002.docx]

S1 Table. Primers sets and corresponding annealing temperatures used for DNA amplification and sequencing.

| Primers name | Position | Sequence 5’ to 3’ | Annealing temperature (^o^C) |
| --- | --- | --- | --- |
| HTSP F1  HTSP R1 | 19-38  1144-1165 | GGCTCAGTAACGGGCGTCTA  ACAGGGACGTATTCATCGCGTC | 58 |
| HTSP F2  HTSP R2 | 839-859  1641-1660 | AGAACACCACAAGGAGTGGA  CCCGTCTTTCCAGAACGGTT | 54 |
| HTSP F3  HTSP R3 | 1330-1350  2008-2027 | CAAGGTAGCTGTAGGAGAACC  TCAGAACAGCTTACTGCCTC | 54 |
| HTSP F4  HTSP R4 | 1819-1838  2695-2714 | AGGTGAAATTGCGCAGGGAG  GTCAACAGACACAGCGCGTT | 58 |
| HTSP F5  HTSP R5 | 2543-2562  3357-3376 | GTGAGAATTCGTCTGGGAGG  GACTGTTGCGCAGTGGTCTT | 56 |
| HTSP F6  HTSP R6 | 3191-3211  3847-3866 | GACGAGATTCCCACTGTCCCT  GCACAATCGCTCCACCAGTG | 54 |
