# Supplementary material for: Description of the Microsporidian Parasite, Heterosporis sutherlandae n. sp., Infecting Fish in the Great Lakes Region, USA
Source: PLoS One. 2015 Aug 5;10(8):e0132027. doi: 10.1371/journal.pone.0132027 (PMC4526549; doi:10.1371/journal.pone.0132027)
Supplement: S2 Table — (DOCX) [file pone.0132027.s003.docx]

S2 Table. Percent identities of *Heterosporis* *sutherlandae* with previously reported fish infecting microsporidians.
