# Supplementary material for: Description of the Microsporidian Parasite, Heterosporis sutherlandae n. sp., Infecting Fish in the Great Lakes Region, USA
Source: PLoS One. 2015 Aug 5;10(8):e0132027. doi: 10.1371/journal.pone.0132027 (PMC4526549; doi:10.1371/journal.pone.0132027)
Supplement: S1 Fig — First position in image represents 600th nucleotide position of the reference sequence. (PDF) [file pone.0132027.s001.pdf]

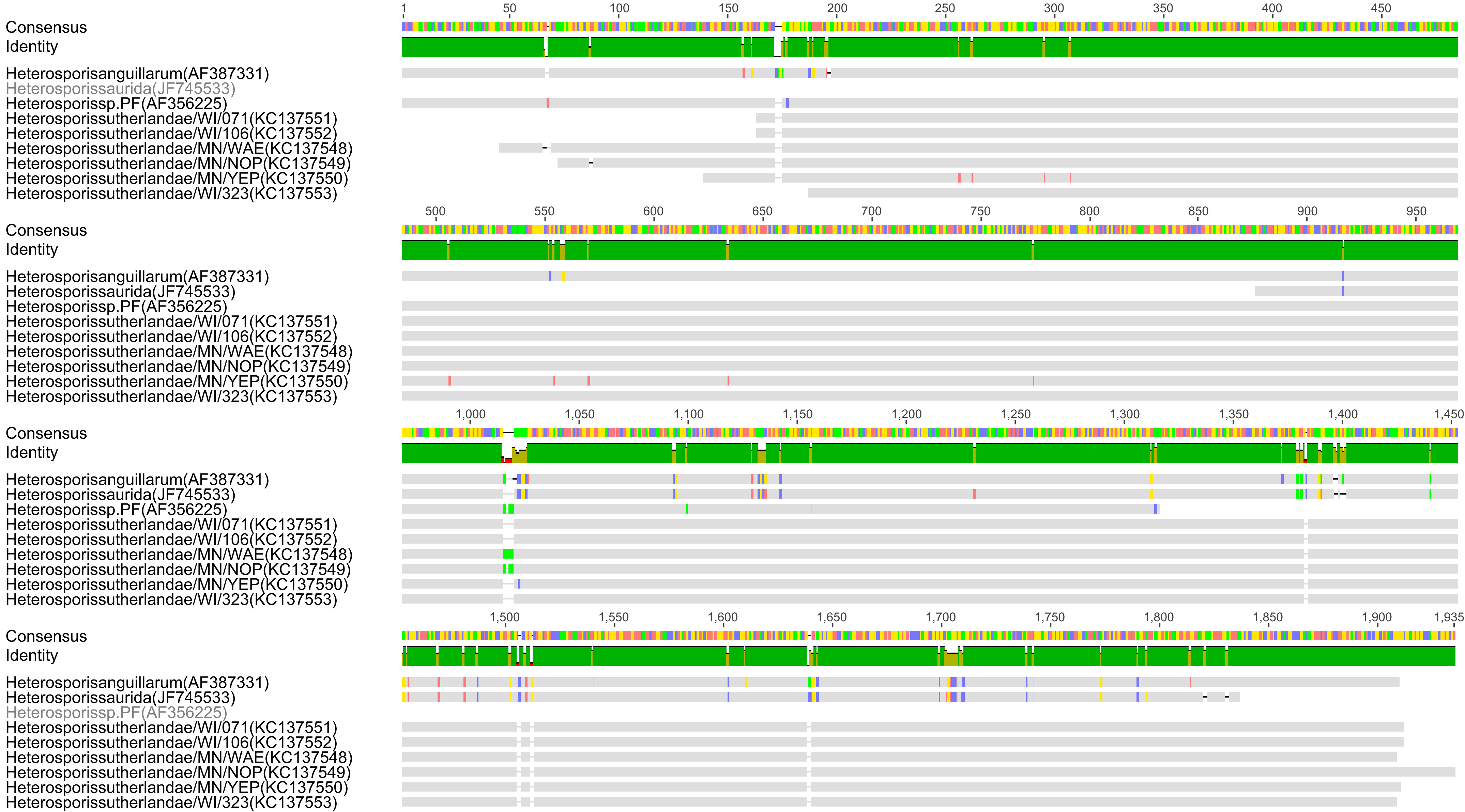
Consensus
Identity
Heterosporisanguillarum(AF387331)
Heterosporissaurida(JF745533)
Heterosporissp.PF(AF356225)
Heterosporissutherlandae/WI/071(KC137551)
Heterosporissutherlandae/WI/106(KC137552)
Heterosporissutherlandae/MN/WAE(KC137548)
Heterosporissutherlandae/MN/NOP(KC137549)
Heterosporissutherlandae/MN/YEP(KC137550)
Heterosporissutherlandae/WI/323(KC137553)
1 50 100 150 200 250 300 350 400 450
500 550 600 650 700 750 800 850 900 950
1,000 1,050 1,100 1,150 1,200 1,250 1,300 1,350 1,400 1,450
1,500 1,550 1,600 1,650 1,700 1,750 1,800 1,850 1,900 1,935
